# Height and overall cancer risk and mortality: evidence from a Mendelian randomisation study on 310,000 UK Biobank participants

**DOI:** 10.1038/s41416-018-0063-4

**Published:** 2018-03-27

**Authors:** Jue-Sheng Ong, Jiyuan An, Matthew H. Law, David C. Whiteman, Rachel E. Neale, Puya Gharahkhani, Stuart MacGregor

**Affiliations:** QIMR Berghofer Medical Research Institute, Brisbane, QLD Australia

**Keywords:** Epidemiology, Risk factors

## Abstract

**Background:**

Observational studies have shown that being taller is associated with greater cancer risk. However, the interpretation of such studies can be hampered by important issues such as confounding and reporting bias.

**Methods:**

We used the UK Biobank resource to develop genetic predictors of height and applied these in a Mendelian randomisation framework to estimate the causal relationship between height and cancer. Up to 438,870 UK Biobank participants were considered in our analysis. We addressed two primary cancer outcomes, cancer incidence by age ~60 and cancer mortality by age ~60 (where age ~60 is the typical age of UK Biobank participants).

**Results:**

We found that each genetically predicted 9 cm increase in height conferred an odds ratio of 1.10 (95% confidence interval 1.07–1.13) and 1.09 (1.02–1.16) for diagnosis of any cancer and death from any cancer, respectively. For both risk and mortality, the effect was larger in females than in males.

**Conclusions:**

Height increases the risk of being diagnosed with and dying from cancer. These findings from Mendelian randomisation analyses agree with observational studies and provide evidence that they were not likely to have been strongly affected by confounding or reporting bias.

## Introduction

Observational studies have shown an association between increased height and cancer risk. The association has been observed for individual cancers such as breast cancer^1^ as well as for overall cancer risk.^2,3^ While these observational studies provide estimates of the extent to which increased height increases cancer risk, these estimates may be biased due to confounding and measurement error. Furthermore, a more fundamental limitation of observational studies is that one cannot determine causality, with confounding and reverse causation^4^ making it difficult to draw conclusions beyond the existence of an association.

Many observational studies are based on self-reported height. These measures are more variable than clinically measured height, and systematic biases exist; for example, people who are older^5^ and shorter tend to overestimate their height, especially men.^6^ Given these biases, observational studies may provide unreliable estimates of the true effect of height on cancer risk. Observational studies find different patterns of association of cancer with height between males and females,^2^ although such findings are potentially adversely affected by mis-reporting.

Mendelian randomisation (MR) is an approach for determining the relationship between a risk factor and an outcome. MR uses the instrumental variable approach, with genetic markers used to form the instrument. An advantage of MR is that, subject to some assumptions, one can gather evidence supportive of a causal relationship between the risk factor and outcome. A number of recent studies have employed the MR approach to investigate the relationship between height and the risk of specific cancers.^7–9^

Here, we use the UK Biobank (UKB) resource to develop a genetic risk score for clinically measured height and use this in an MR framework to infer a causal relationship between height and cancer risk. Our primary focus is on whether height predicts 'any cancer' occurrence in UKB—such an outcome variable is intrinsically interesting because it represents the outcome 'will I get any cancer by age ~60' (UKB participants have median year of birth 1950, with cancer registry data complete to at least the end of 2011). Further, we use death registry data to assess how height affects cancer mortality by age 60.

## Methods

### Description of the UK Biobank—genotyping quality control, ancestry

The UKB is a large population-based cohort consisting of 502,649 participants (recruited during 2006–2010) aged between 37 and 73 years old living in the United Kingdom. Each participant completed a series of baseline assessments at one of 22 assessment centres across the United Kingdom, including physical assessments and face-to-face interviews on medical conditions. Participants were also genotyped either via the Affymetrix UK BiLEVE Axiom array or the Affymetrix UK Biobank Axiom array. Imputations were done against the UK10K,^10^ 1000 Genomes Phase 3^11^ and Haplotype Reference Consortium (HRC) reference panels.^12^ Specific detail of the genotyping QC was described elsewhere.^13^ Imputed SNPs were retained for analysis if MAF > 0.001, Imputation INFO score >0.6, and are present in the HRC panel. Among the 502,649 participants, we restricted analysis to a set of 438,870 participants (see supplementary Fig 1) of white-British ancestry who passed genotyping Quality Control. We accounted for genetic relatedness between individuals as described below.

### Phenotype cleaning for UK Biobank height, and cancer outcomes

There are various phenotypic definitions of height in the UKB study. Here we used the Seca 202 measured standing height (UKB Field-ID:50). For those with repeated measurements, the average was used. We performed a GWAS on height using the BOLT-LMM v2.3 package, which accounts for cryptic relatedness within the sample, allowing related individuals to be retained, maximising power. Total of 360,087 randomly selected genotyped SNPs sparsely distributed over the genome were used to infer the structure within the sample, correcting for both the ancestry structure and the relatedness in UKB. We further excluded cancer cases (*n* = 46,531) from the height GWAS analyses to avoid bias from reverse causality. In brief, GWAS was performed on standing height for 391,029 individuals adjusting for age and genetic sex. For the MR analyses, only variants (filtered for MAF >0.05) that are associated with height at a *P* value <1 × 10^−8^ were used as instruments. Prior to our MR analyses, SNPs associated with height were pruned at *r*^2^ = 0.01 using a 10 Mb window to ensure independence among instruments.

Cancer phenotypes were collated based on cancer registry records with cases defined based on any presence of ICD10 cancer ('C') code entries, except C44 (other malignant neoplasms of skin). More precisely, individuals diagnosed with cutaneous squamous cell carcinoma or basal cell carcinoma (BCC) (ICD10 code C44), or who self-reported having had cancer but where no confirmation was obtained, were excluded from the analysis. Healthy controls were defined as any individual without cancer, benign or in situ tumour (including C44) recorded in the cancer registry, and must have had no self-reported history of cancer. The distribution of age at last visit/diagnosis between cases and controls were shown in Supplementary Figure 2. The complete case/control selection procedure is given in Table 1. We further excluded related people using a hierarchical approach to maximise sample size using the --rel-cut-off feature in PLINK (v2.00 alpha; available at https://www.cog-genomics.org/plink/2.0)^14^ at a π̂-threshold of 0.2. First, we excluded related samples within cases and within controls separately. For π̂ > 0.2 relationships between cases and controls, we selectively retained cases using a pairwise relatedness matrix generated through --genome in PLINK (v2.0 alpha). The only exception was in the very rare situation where a case was related to >4 controls; in such situations the case was dropped. The exclusion and trimming procedures on genetic relatedness are illustrated in Supplementary Figure 3. Our final sample size for the cancer risk MR study was 264,638 healthy controls and 46,155 cancer cases.

**Table 1:** Inclusion criteria for case and control definition for overall cancer risk and mortality MR study

UKB field ID	Description	Case/control criteria
134	(Number of self-reported cancers)	If there is at least one question with a value >0, then it is excluded from the control set
2453	(Cancer diagnosed by doctor Medical conditions)	
20001	(Cancer code, self-reported)	If there is at least one question with a value which is not 'NA', then it is excluded from the control set
20007	(Interpolated age of participant when cancer first diagnosed Medical conditions) as above	If there is at least one question with a value which is not 'NA', then it is excluded from the control set
40001	(Underlying (primary) cause of death: ICD10—Death register)	If its ICD10 code starts with 'C' or 'D' then exclude from the control set. If the ICD10 code starts with 'C' but is not 'C44' (i.e., not BCC/SCC cases), then it is classified as a case
40006	(Type of cancer: ICD10—Cancer register)	If its ICD10 code starts with 'C' or 'D' then exclude from the control set. If the ICD10 code starts with 'C' but is not 'C44' (i.e., not BCC/SCC cases), then it is classified as a case
40007	(Age at death—Death register)	If there is at least one question with a value which is not 'NA', then it is excluded from the control set
40008	(Age at cancer diagnosis—Cancer register)	If there is at least one question with a value which is not 'NA', then it is excluded from the control set
40011	(Histology of cancer tumour—Cancer register)	If there is at least one question with a value which is not 'NA', then it is excluded from the control set
40012	(Behaviour of cancer tumour—Cancer register)	If there is at least one question with a value which is not 'NA', then it is excluded from the control set
40013	(Type of cancer: ICD9—Cancer register)	If there is at least one question with a value which is not 'NA', then it is excluded from the control set
84	(Cancer year/age first occurred—Medical conditions)	If there is at least one question with a value which is not 'NA', then it is excluded from the control set
40009	(Reported occurrences of cancer—Cancer register)	If there is at least one question with a value >0, then it is excluded from the control set
40006	(Type of cancer: ICD10—Cancer register)	If its ICD10 code starts with 'C' or 'D' then exclude from the control set. If the ICD10 code starts with 'C' but is not 'C44' (i.e., not BCC/SCC cases), then it is classified as a case
41202	(Diagnoses—main ICD10—Summary Information (diagnoses))	
41204	(Diagnoses—secondary ICD10—Summary Information (diagnoses))	
	Summary of procedure: Control	Individuals did not report any instance of cancer diagnosis—whether through self-report or clinical diagnosis
	Summary of procedure: Cases	Individuals reported instances of cancer diagnosis validated through ICD10 codes starting with 'C'. However, individuals with C44 were excluded

Caption: Similar criteria were used for the cancer mortality study, mainly we additionally included field ID 40001 and 40002 to validate that cause of death for participants in the study were cancers

Among the 14,417 deceased participants in UKB, 7348 reportedly died from cancer (ICD10 cancer definitions as above) according to the UK Death Registry. UKB individuals with mismatched entries between UK cancer registry and Death registry on cancer status were removed. Relatedness in the mortality analysis was managed as above, resulting in 270,342 healthy controls and 6998 deceased cancer cases. For both overall cancer risk (controls vs. all cancer cases), risk of specific cancer types (defined by ICD10 groups, see Supplementary Table 1) and overall cancer mortality (controls vs. people who died from cancers), we fitted a GWAS logistic regression model using PLINK v2.00 alpha. We fitted the first 10 ancestral principal components computed by UKB, genetic sex and age as covariates in the model.

### Instrumental variable analyses

For an MR experiment to be valid, the following assumptions have to be satisfied. First, the genetic instrument used has to be robustly associated with the exposure of interest (i.e., height). Second, the genetic instrument cannot be associated with any confounders. Lastly, the genetic instrument can only be associated with the outcome through the exposure. We used the two-sample MR approach, where the associations between SNPs and height and SNPs and cancer outcomes can be estimated separately. Our MR causal estimate was estimated via the Wald-type ratio estimator,^15^ which is an inverse variance-weighted model combining the estimates of every height SNP instruments on cancer. The causal odds ratio estimated from the Wald-type estimator represents the odds of cancer (incidence or mortality) per centimetre increase in genetically predicted height.

The TwoSampleMR and MendelianRandomization R packages^16,17^ were used to test for MR assumption violations. By design, the first assumption was trivially satisfied as we only adopted genetic instruments that were clearly associated with height at a *P* value <1 × 10^−8^ (clearly in excess of typical strong instrument definition for MR and also significant after accounting for multiple testing of SNPs genome-wide). Palindromic SNPs with strands that cannot be inferred via effect allele frequency information were also dropped from the analyses. We applied MR Egger regression and weighted median models to assess whether causal estimates were influenced by horizontal pleiotropy and bias from a proportion of invalid instruments. We tested for bias due to association of SNP with potential confounders by recomputing the causal estimate with a subset of SNP instruments that are not associated with any potential confounder on cancer.

## Results

We used a total of 2059 independent genetic variants as instruments for standing height, explaining ~11% of the phenotypic variance. For a one standard deviation (SD), 9.27 cm increase in genetically determined standing height, the estimated causal odds ratio (COR) on overall cancer risk was 1.098 (95% confidence interval (CI): 1.065–1.132). The magnitude of association was lower in males with a COR of 1.055, (95% CI: 1.012–1.100) than in females (COR 1.139, 95% CI: 1.093–1.186). The COR was significantly lower in males than in females (*P* = 0.01). The causal estimates for each individual SNP instrument on cancer outcomes are provided in (online) Supplementary material.

The COR estimates based on individual cancer types are given in Supplementary Table 1 and Figure 2. In brief, the direction of effect for most cancer types were consistent with the overall cancer MR analyses. However, the COR estimate for prostate cancer (COR 0.998, 95% CI: 0.918–1.086) suggests a null relationship with height, whereas the estimates for stomach, oesophageal (COR 0.946, 95% CI: 0.806–1.109) and pancreatic cancer (COR 1.075, 95% CI: 0.858–1.347) were too wide to make any meaningful causal inference.

**Fig. 1:**
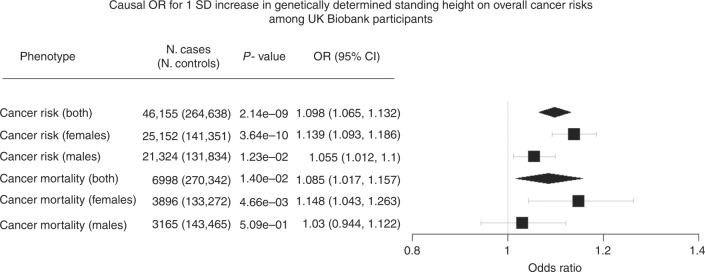
Mendelian randomisation estimate for height on overall cancer risk and cancer mortality. N. cases and N. control refer to the number of cases and controls in each analyses. The number of cases/controls for each sub-analysis differ due to different distribution of relatedness in the effective sample

**Fig. 2:**
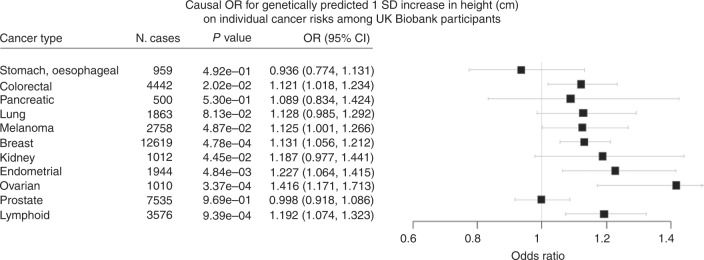
Mendelian randomisation estimates for the association between height and individual cancer types. N. cases refer to the number of cases present in each cancer type

The estimated COR for a 1 SD increase in height on cancer mortality was 1.085 (95% CI: 1.017–1.157; Fig. 1). However, the association was mainly driven by females (COR 1.148, 95% CI: 1.043–1.263) as the association did not meet statistical significance for males (COR 1.030, 95% CI: 0.944–1.122). The male COR was not significantly different from the female COR (*P* = 0.097) (Fig. 2).

Additional sensitivity analyses were performed to ensure that our association estimates were not biased by violation of the MR assumptions. There was no evidence that our estimates were influenced by directional pleiotropy (MR Egger intercept *P* value >0.05, Supplementary Table 2). In short, both the penalised weighted median and Egger regression yielded concordant estimates. Furthermore, to minimise the risk of SNP-confounding issues, we repeated our MR experiment using height SNPs that are not associated with any potential cancer confounders (smoking, BMI, coffee/tea intake, alcohol). The corresponding Wald-type COR estimate based on the filtered 1267 SNPs was 1.084 (1.042–1.127), showing that our original estimates are unlikely to have been biased by these confounders. Results of the alternative MR methods explored are summarised in Supplementary methods and Supplementary Figure 4.

We performed stratified analyses to investigate whether the association between height and cancer had a different effect size among smokers. When stratified by smoking status (ever vs. never-smokers), the magnitude of the association for a 1 SD increase in genetically predicted height on cancer was higher in female non-smokers than in female smokers (COR 1.178; 95% CI: 1.112–1.248 vs. COR 1.109; 95% CI: 1.054–1.167) although the difference was not statistically significant (*P* = 0.127). A similar pattern of association was observed in the cancer mortality analysis, where the estimated COR was higher in female non-smokers. We did not observe any meaningful difference when stratifying by smoking status among males. Results based on stratifying smoking status are given in Supplementary Table 3.

## Discussion

Here, we have used Mendelian randomisation to estimate causal associations between height and cancer risk and mortality. We used a large-scale biobank (UKB) to derive accurate estimates of the causal effect of height, with our MR approach yielding estimates which are likely to be unaffected by the biases which frequently affect observational studies (confounding, reverse causality, measurement error/bias).

The estimates we have derived are concordant with those estimated from well-designed observational studies; a meta-analysis of previous observational studies estimated that a 10 cm increase in height was associated with an increased risk of cancer in men (OR 1.10, 95% CI: 1.08–1.12) and women (OR 1.15, 95% CI: 1.14–1.17).^2^ Similarly, an observational study using the EPIC cohort showed an association between height and increased cancer mortality, with a larger effect seen in women than in men.^18^ Another large observational study (Million Women study) showed a larger effect of self-reported height on cancer risk in women who were non-smokers (1.19 in non-smokers, 1.11 in current smokers).^2^ Our MR-based estimates yielded similar estimates, although our findings for the difference between female non-smoker vs. smoker COR did not reach significance.

Previous MR studies have examined specific cancers. Our findings are concordant with a previous MR study focusing on prostate cancer where they found no effect of height on cancer risk but did find an effect on mortality, albeit only in low-grade disease.^19^ In breast cancer, an MR study found that a 10 cm height increase conferred an OR of 1.22;^9^ this is larger but not significantly different to our estimate for breast cancer (COR = 1.13). A previous MR study of colorectal cancer^8^ found that a 10 cm increase in height conferred an OR of 1.07 (95% CI: 1.01–1.14) for risk, with a larger effect in women.

There are several notable strengths of our MR study. First, the UKB participants used in this study were all verified through genetic measures to be of white-British ancestry (Supplementary Figure 1), ensuring that our study population is homogeneous. The genetic instruments used for height explained about 15% of the phenotypic variance among Europeans, allowing for well-powered MR analyses. Our use of a consistent ICD10 definition of cancer diagnosis and cancer mortality enables better replication of our findings and allows meaningful comparisons against other studies investigating individual cancer types. Sensitivity analyses further revealed that our causal estimates were not biased by weak violation of MR assumptions nor statistical specificity of MR methods (see Supplementary Table 2).

However, there are several limitations to be considered in interpreting our MR findings. Despite our effort to carefully define healthy controls for the MR analyses, the overall cancer phenotype is a compilation of various types of ICD10-defined cancers and hence is by construction rather heterogeneous. The distribution of individual cancer cases based on the demography of middle aged (40–65) Europeans might underestimate the population prevalence for cancers with late onset. This decreases the power for the individual cancer MR analyses in the UKB; however, the large variance explained by our genetic instruments helps circumvent this issue.

In our MR analysis, there is an intrinsic assumption that the relationship between height and the log(OR) on cancer risk/mortality is strictly linear. As height-promoting alleles on average predispose a very small additive increase in height relative to the population, MR studies are only able to assess a (linear) change in height on cancer risk in a population. These findings are not necessarily comparable to studies investigating putatively non-linear relationships between height and cancer at tail-ends of the height distribution. Hence, the MR estimate of the COR can be interpreted as the averaged change in cancer risk for a standard deviation increase in height.

Another potential issue is the use of Wald-type ratio estimators to estimate the causal association. The Wald-type ratio is a widely used estimator among 2-sample MR studies in which individual level data may be unavailable for conventional instrumental variable techniques. It has been widely discussed that estimation of SNP-exposure and SNP-outcome effects within the same sample are known to induce bias due to winner’s curse and reverse causation.^20^ However, this is unlikely to have substantially biased our results as we made the following adjustments. First, we attempted to remove winner’s curse bias via setting a more stringent threshold^21^ for instrument validation (height *P* < 1 × 10^−8^). Furthermore, in our analyses we only estimated the SNP-height association among cancer-free individuals, and hence our estimates are unlikely to have been subject to bias and inflated type I error, as discussed elsewhere.^20^ Our sensitivity analyses (Supplementary Table 2) showed limited evidence for bias due to pleiotropy. Lastly, in contrast to some previous studies, we derived a new SNP instrument from the UK Biobank data rather than using previously published SNPs associated with height.^22^ We did this because (i) height in UK Biobank was clinically measured, avoiding self-reporting biased, (ii) after our quality control, the UK Biobank set represented a large homogeneous sample set, (iii) a small proportion of the previously reported height SNPs did not clearly replicate in UK Biobank. As a robustness check, we recomputed our MR estimates using SNP instruments (at *P* < 1 × 10^−8^) obtained from the publicly available^22^ height GWAS (Supplementary Table 4); Our findings were broadly unchanged.

SNP-pleiotropy is an essential limitation to address in every MR experiment. Horizontal pleiotropy refers to the scenario, where the SNP instrument used is independently (in parallel) associated with the outcome via a different biological mechanism to the exposure of interest. Conceptually, MR methods like the MR Egger regression^23^ and the penalised weighted median model^24^ can reduce the estimate bias in the presence of weak violation of MR assumptions. To further evaluate the bias due to pleiotropy, we also repeated the MR analyses removing any height instruments associated with potential confounders (smoking, BMI, coffee consumption, alcohol intake). Our results were essentially unchanged following these removals, suggesting that our causal inference was robust.

We have provided genetic evidence for an association between height and cancer risk; however, it is beyond the scope of these analyses to disentangle the biological mechanism behind this relationship. It has previously been postulated that the increased risk conferred by height is attributable to more cells in taller compared with shorter people. In addition, height is a highly polygenic trait, with many biological pathways implicated in determining variation in height (e.g., skeletal growth, FGF signalling, WNT signalling, regulation of beta-catenin, mTOR signalling).^22^ Understanding the causal biological pathways between genetic variants that contribute to height, and subsequently cancer risk, requires functional annotation of variants, along with larger sample sizes to achieve sufficient statistical power for these analyses.

## Conclusion

In conclusion, we have demonstrated that increased genetically determined height is causally associated with overall cancer susceptibility as well as with greater cancer mortality by age 60 among white Europeans (British). Future studies are necessary to elucidate the specific biological mechanisms which underlie the association between height and cancer.

## Electronic supplementary material

Supplementary material(DOCX 1799 kb)

Supplementary Figure 1(TIF 947 kb)

Supplementary data (excel spreadsheet)(XLSX 1001 kb)
