# Supplementary material for: Height and overall cancer risk and mortality: evidence from a Mendelian randomisation study on 310,000 UK Biobank participants
Source: Br J Cancer. 2018 Mar 27;118(9):1262–7. doi: 10.1038/s41416-018-0063-4 (PMC5943400; doi:10.1038/s41416-018-0063-4)
Supplement: Supplementary file 1 — Supplementary material(DOCX 1799 kb) [file 41416_2018_63_MOESM1_ESM.docx]

Supplementary material for manuscript “***Association of height and overall cancer risk: evidence from mendelian randomization study on 310,000 UK Biobank participants”***

**Supplementary methods:**

Wald-type ratio MR estimator:

Using only summary statistics, the magnitude of association for genetically inferred one unit change of the exposure (height) on the outcome (cancer) can be evaluated via

$$\hat{\beta}_{IVW}=\frac{\sum_{z} \hat{\beta}_{zx}\hat{\beta}_{zy}\sigma_{zy}^{-2}}{{\sum_{z} \hat{\beta}_{zx}^{2}}\sigma_{zy}^{-2}}$$

$$\sigma_{IVW}=\sqrt{\frac{1}{\sum_{z} \hat{\beta}_{zx}^{2}\sigma_{zy}^{-2}}}$$

where $\hat{\beta}_{IVW}$ and $\sigma_{IVW}$ represents the inverse-variance weighted (wald-type ratio) estimator and its corresponding standard error. Here $\hat{\beta}_{zy}$ and $\sigma_{zy}$ refer to the log(OR) of the SNP instrument (Z) with cancer outcome (Y) and its corresponding standard error. $\hat{\beta}_{zx}$ is the magnitude of association of the SNP instrument (Z) with height in cm (X).

**Sensitivity analyses**All of the sensitivity MR analyses were carried out using the TwoSampleMR package written in R. Each alternative MR methods presented were performed using summary statistics only. Technical details and model specification of each of the MR methods evaluated are available in their respective publications. Results for the sensitivity analyses were provided in Supplementary Figure 3.

**Subsetting confounder-free height SNPs**In total, 2051 SNPs were used for the MR analyses. To evaluate whether our MR association were potentially driven by SNP-confounder bias, we filtered all of our SNP of interest such that the filtered set does not contain variants associated (P<0.01) on any of the following potential confounders: alcohol intake, coffee/tea intake, smoking status, smoking cigarette per day, and body mass index. Our filtered SNP set contain 1,267 height SNPs.

**Supplementary Table 1: Criteria for cancer type classification based on ICD10 diagnosis.**

| **Cancer type grouping** | **ICD Groups** | **ICD10 prefix of cancer type** | **Comments** |
| --- | --- | --- | --- |
| Stomach, esophageal | C15-C26 | C15,C16 |  |
| Colorectal | C15-C26 | C18,C20,C21 |  |
| Pancreatic | C15-C26 | C25 |  |
| Lung | C30-C39 | C34 |  |
| Melanoma | C43-C44 | C43 |  |
| Breast | C50-C50 | C50 | Females only |
| Kidney | C64-C68 | C64 |  |
| Endometrial | C51-C58 | C53,C54 | Females only |
| Ovarian | C51-C58 | C56 | Females only |
| Prostate | C60-C63 | C61 | Males only |
| Lymphoid | C81-C96 | C81-C96 |  |

Grouping of cancer cases were based on ICD10 diagnosis codes from UKB data-field (40006,41202,41204). For individuals diagnosed with multiple ICD10 cancer codes, the individual will be indexed in all of the cancer types he/she is diagnosed with.

**Supplementary Table 2: Estimates of Egger intercept to evaluate evidence for directional pleiotropy in MR association.**

| **Outcome** | **Exposure** | **Egger_intercept** | **se of Egger_intercept** | **Pval** |
| --- | --- | --- | --- | --- |
| overall cancer risk | height | -0.00052858 | 0.000499746 | 0.290 |
| overall cancer risk (males) | height | -0.000321798 | 0.000681506 | 0.637 |
| overall cancer risk (females) | height | -0.000705531 | 0.000663815 | 0.288 |
| overall cancer mortality | height | -0.00040018 | 0.001058414 | 0.705 |
| overall cancer mortality (males) | height | -0.000784098 | 0.001409444 | 0.578 |
| overall cancer mortality (females) | height | 3.45E-05 | 0.001563413 | 0.982 |

Here, pval refers to the pvalue of the estimated Egger intercept being null. A significant (P<0.05) pvalue would present evidence that the MR causal estimates derived via the inverse-variance weighted model incorporating directional pleiotropy.

**Supplementary Table 3: MR Association of genetically predicted height on cancer risk and mortality among smokers.**

| **Outcome** | **Causal OR per 1SD increase in height** | | | | |  |  |  |
| --- | --- | --- | --- | --- | --- | --- | --- | --- |
|  | ***Among never-smokers*** | |  | ***Among ever-smokers*** | |  | **ChiSq of diff.** | **Pvalue of diff.** |
|  | **COR(95% CI)** | **Pval** |  | **COR(95% CI)** | **Pval** |  |  |  |
| overall cancer risk (both) | 1.110(1.060,1.162) | 9.46E-06 |  | 1.092(1.052,1.133) | 3.60E-06 |  | 0.29 | 0.587 |
| overall cancer risk (Females) | 1.178(1.112,1.248) | 3.09E-08 |  | 1.109(1.054,1.167) | 6.73E-05 |  | 2.32 | 0.127 |
| overall cancer risk (Males) | 1.007(0.935,1.084) | 0.855 |  | 1.080(1.028,1.135) | 0.002 |  | 2.40 | 0.122 |
|  |  |  |  |  |  |  |  |  |
| overall cancer mortality (both) | 1.125(1.002,1.262) | 0.046 |  | 1.061(0.983,1.146) | 0.131 |  | 0.68 | 0.409 |
| overall cancer mortality (Females) | 1.258(1.079,1.465) | 0.003 |  | 1.082(0.959,1.221) | 0.200 |  | 2.29 | 0.130 |
| overall cancer mortality (Males) | 0.968(0.811,1.156) | 0.721 |  | 1.046(0.948,1.155) | 0.369 |  | 0.56 | 0.453 |

**Supplementary Table 4: Comparison of MR Association of genetically predicted height on cancer risk and mortality for UK Biobank height instruments and instruments from Wood et al. (2014)**

|  | **Based on UKB height GWAS (controls only) (height p<1e-8)** | | | | | **Based on Wood et al. height GWAS  (height p<5e-8)** | | | |
| --- | --- | --- | --- | --- | --- | --- | --- | --- | --- |
| **Outcome** | **P-value** | **COR** | **CI_lower** | **CI_upper** |  | **P-value** | **COR** | **CI_lower** | **CI_upper** |
| Overall cancer risk | 2.14E-09 | 1.1 | 1.06 | 1.13 |  | 1.21E-07 | 1.08 | 1.05 | 1.11 |
| Overall cancer risk (Females) | 3.64E-10 | 1.14 | 1.09 | 1.19 |  | 1.93E-07 | 1.1 | 1.06 | 1.15 |
| Overall cancer risk (Males) | 0.01233 | 1.05 | 1.01 | 1.1 |  | 0.00745 | 1.06 | 1.01 | 1.1 |
| Overall cancer mortality | 0.01399 | 1.08 | 1.02 | 1.16 |  | 0.00025 | 1.12 | 1.05 | 1.19 |

Note: The SNP instruments for height based on the Wood et al. 2014 GWAS was obtained from MR-Base (http://www.mrbase.org/).

**Supplementary Figure 1: Plot of ancestral principal component values (PC1-PC2) in UK Biobank participants (N=487,910)**

[See Supplementary plot (online material)]

Caption: The red eclipse represent the bound of PC1-PC2 values based on self-reported white-British participants. Samples with PC1-2 values within the eclipse are hence categorised as individuals of white-British ancestry for our analyses.

**Supplementary Figure 2: Relatedness-trimming procedure for SNP-cancer association**


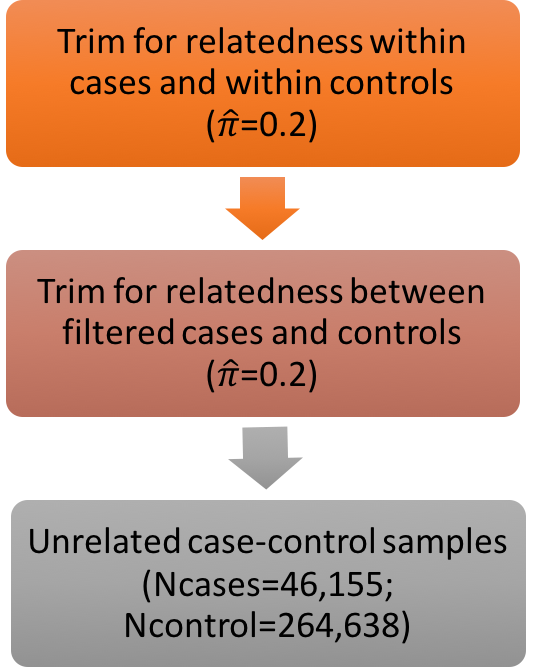


**Supplementary Figure 3:** **Distribution of Age at last follow-up for UK Biobank participants**


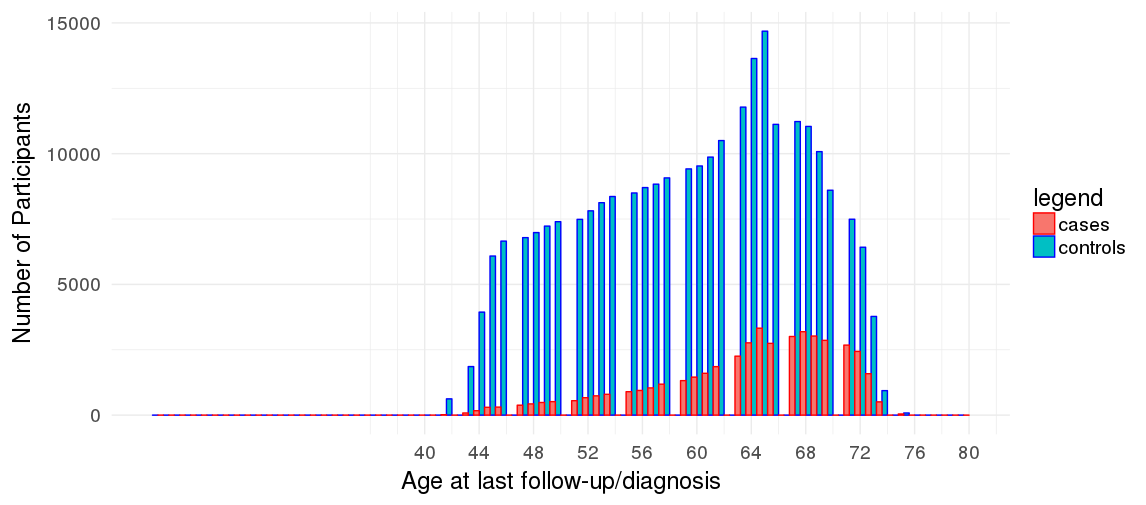


**Supplementary Figure 4: Assessment of MR association between height and overall cancer risk using alternative MR methods**


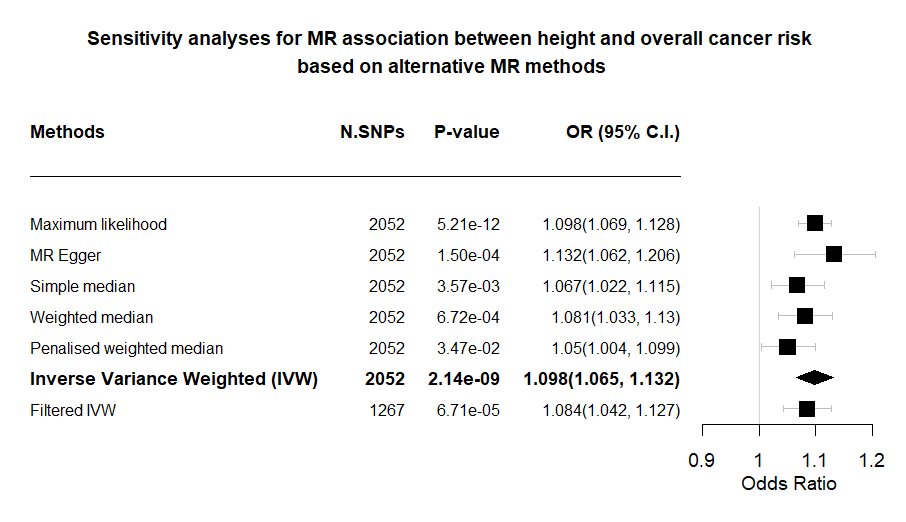


*Method highlighted in bold was used for the main analyses. Magnitude of association presented are based on one SD increase in genetically predicted height.
