# Supplementary figures and images for: Height and overall cancer risk and mortality: evidence from a Mendelian randomisation study on 310,000 UK Biobank participants
Source: Br J Cancer. 2018 Mar 27;118(9):1262–7. doi: 10.1038/s41416-018-0063-4 (PMC5943400; doi:10.1038/s41416-018-0063-4)

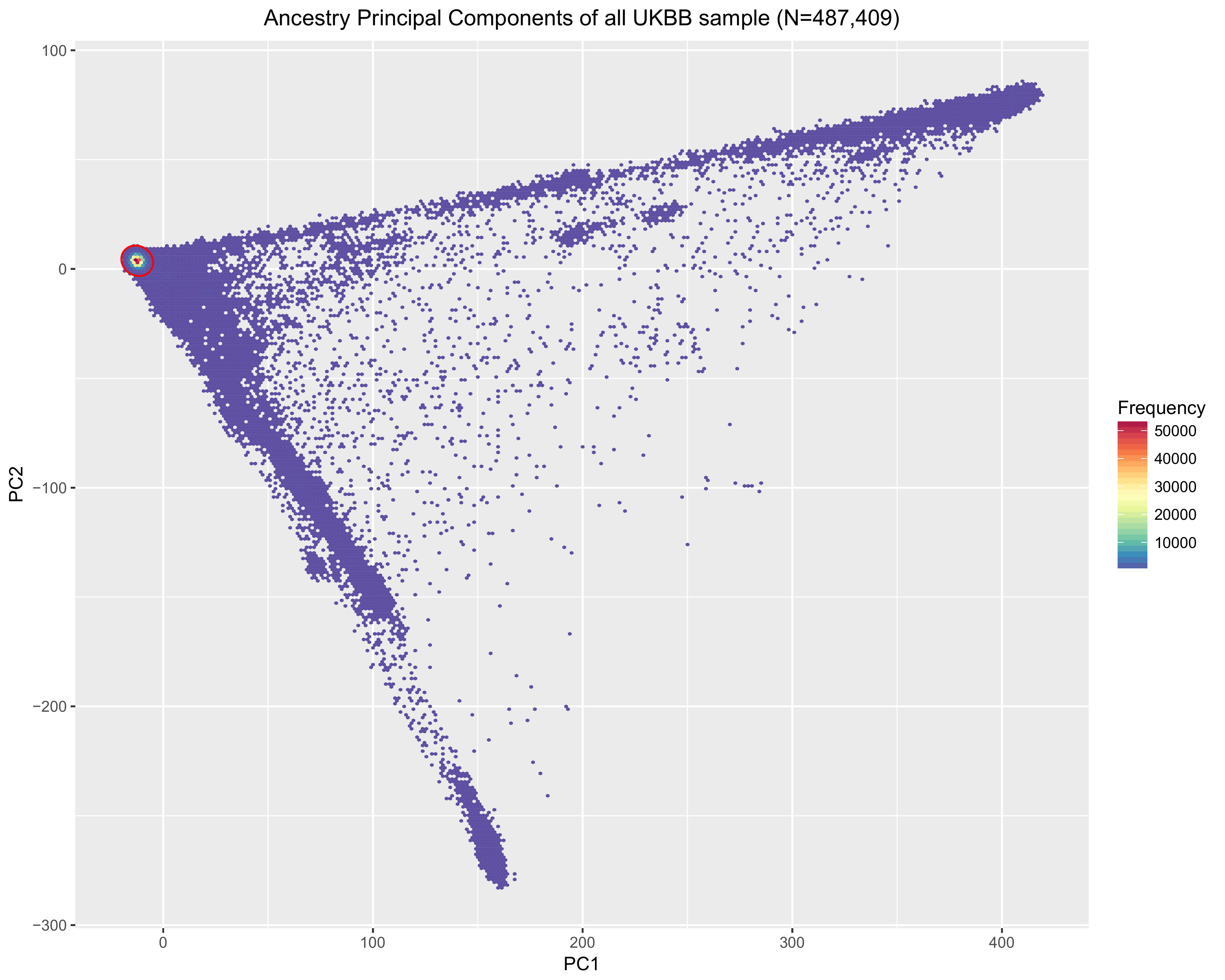

Supplement: Supplementary file 2 — Supplementary Figure 1(TIF 947 kb) [file 41416_2018_63_MOESM2_ESM.tif]
